# Co-exposure of dimethomorph and imidacloprid: effects on soil bacterial communities in vineyard soil

**DOI:** 10.3389/fmicb.2023.1249167

**Published:** 2023-11-02

**Authors:** Jean Chang, Fo-Ting Shen, Wei-An Lai, Chien-Sen Liao, Wen-Ching Chen

**Affiliations:** ^1^International Master Program in Agriculture, National Chung Hsing University, Taichung, Taiwan; ^2^Department of Soil and Environmental Science, National Chung Hsing University, Taichung, Taiwan; ^3^Innovation and Development Center of Sustainable Agriculture (IDCSA), National Chung Hsing University, Taichung, Taiwan; ^4^Department of Medical Science & Biotechnology, I-Shou University, Kaohsiung, Taiwan; ^5^International Bachelor Program in Agribusiness, National Chung Hsing University, Taichung, Taiwan

**Keywords:** morpholine fungicide, neonicotinoid insecticide, non-target organisms, relative microbial abundance, ecological functions

## Abstract

In Taiwan, the pesticides dimethomorph and imidacloprid are recommended for pest control in vineyards. Therefore, tank-mixing of these two pesticides is usually a routine practice before application. This study analyzed the influence of vineyard soil microbial flora under the recommended and high dosages (100 times the recommended dosage) of dimethomorph and imidacloprid. Individual and combined applications of pesticides were also tested through batches of soil incubation experiments. Four treatments—control (C), dimethomorph (DT), imidacloprid (IM), and mixed application of dimethomorph and imidacloprid (ID)—were used in the experimental design. From the soil metabolism, no significant reaction was observed after 2 months in the recommended dosage group, regardless of whether the pesticides were being applied individually or combined. For the high dosage, imidacloprid showed a higher effect than the co-exposure treatments, showing a possible prolonged effect after its repetitive application. From PCoA analysis, pesticide treatments altered the soil ecology after 2 months, and the effect of imidacloprid can be explicitly observed at high dosages. At the phylum level, *Acidobacteria* can indicate pesticide application around the recommended dosage. It was inhibited by ID on day 7 and was augmented by all pesticides on day 63. The effect of the recommended dosage of pesticide mixtures after 2 months of incubation was revealed in the minor families *Gemmataceae* and *Pirellulaceae*, while the high dosage treatments affected both the core and the minor families. Our findings verified the changes in the composition of microbial communities upon pesticide application, which would affect carbon, nitrogen, sulfur, phosphorous cycles, and contaminant removal ability within the vineyard.

## 1. Introduction

In the process of agricultural production, to save labor and effort, different pesticides can be mixed before application. In Taiwan, dimethomorph and imidacloprid are both recommended in grape production (Ministry of Agriculture, 2018), and the mixed application of the two pesticides is often found. In addition, to effectively control pests and diseases such as thrips and downy mildew, fungicides and insecticides can be applied weekly in the vineyard during the growing season until the required pre-harvest intervals are due (Pers. Commun.).

The neonicotinoid systemic insecticide imidacloprid {1- [(6- chloropyridin- 3- yl) methyl]- N- nitro- 4, 5- dihydroimidazol- 2- amine} has relatively high water solubility (0.61 gL^−1^) and is therefore considered to have high groundwater pollution potential (Flores-CéSpedes et al., 2012). It acts on the central nervous system of insects such as thrips, termites, and fleas (Magalhaes et al., 2009). It is widely used in agriculture production (Gervais et al., 2010; Wang et al., 2023). However, imidacloprid can persist without sunlight, with a half-life of 3 years (Bonmatin et al., 2015).

Dimethomorph is a cinnamic acid derivative and is a member of the morpholine chemical family. The fungicide inhibits fungal cell wall synthesis, leading to the death of fungal cells. In a study by Liang et al. (2011), dimethomorph had an estimated half-life of 11.5–18.5 days following a first-order kinetic degradation formulation. However, due to its high hydrolytic stability, dimethomorph can remain stable for up to 5 years without exposure to sunlight.

Many studies have shown that the mixed application of pesticides has adverse effects on soil-borne organisms. A meta-study examined 394 studies and concluded that negative effects, such as lower reproduction rates, higher mortality rates, or changes in behaviors, were found in 70.5% of the 2,842 tested parameters in soil invertebrates (Gunstone et al., 2021). It also revealed a reduction in microbial diversity and abundance after pesticide application in paddy rice fields (Onwona-Kwakye et al., 2020). A global-scale geostatic study found the highest pesticide mixture contents in orchard and grape cropping systems (Tang and Maggi, 2021). The co-exposure of pesticides such as dimethomorph and imidacloprid is a critical issue, especially in grape vineyards.

Agricultural practices in the field causing pesticide exposure in soils may disturb the sensitive balance of microflora, affecting the soil's nutrient cycles and fertility (Prashar and Shah, 2016). The changes in microbial communities and activities have often been observed as indicators for the degree of effect on various agricultural inputs, such as pesticides (Gangola et al., 2021, 2022b). A study assessed bacterial communities to utilize the sensitive nature of some bacteria as biomarkers of heavy metal contamination (De La Rosa-Acosta et al., 2015). Burns et al. (2013) measured soil enzyme activities to indicate microbial diversity and soil quality. Gianfreda and Rao (2008) observed the changes in soil microbial activity and biomass induced by pesticides. Microorganisms may be an excellent indicator of soil health change, quality improvement, or soil degradation (Mahdi et al., 2017). The study aims to investigate the effect of dimethomorph and imidacloprid applied at the recommended application rate and high dosages individually and as a mixture on soil microflora in the vineyard soil.

## 2. Material and methods

### 2.1. Soil sampling and soil physicochemical properties

Soil samples were taken from the top layer (0–20 cm) at bare land areas in a vineyard located at Taichung District Agricultural Research and Extension Station, Taiwan (24°00'04.0”N 120°32'04.7”E). The soil was collected by shovels after the grapes were harvested for 2 weeks. Plant protection products or fertilizers were added during this period. The soil was sieved (2 mm) and air-dried at room temperature for a week before the study.

The soil properties were determined as described in brief: air-dried soil samples were analyzed for pH and electrical conductivity (EC) at the soil-to-water ratio of 1:2 (Multi 9620 IDS, W.T.W., Germany). Soil-available nitrogen was determined using the Kjeldahl method (Bremner, 1960). The soil organic matter and soil texture were determined using the dry combustion method (Davies, 1974) and the hydrometer method (Bouyoucos, 1936), respectively. Soil available phosphorus was determined using the Bray-1 method at a wavelength of 650 nm (Thermo Scientific GENESYS™ 30 Visible Spectrophotometer). Soil exchangeable potassium was determined using flame atomic adsorption spectrophotometry (ICP-AES; PerkinElmer Avio200, Waltham, MA, USA).

The pH of the soil was 6.03, and the organic matter content of loamy sand was 19.1%. Before the treatments, the available N, P, and K were 257.8, 7.81, and 146.5 mg kg^−1^, respectively.

### 2.2. Pesticide treatments and analysis

The pesticide products of 28.8% SL formulation of imidacloprid (Great Victory Chemical Industry Co., LTD) and 50% SC formulation of dimethomorph (Chia Tai Enterprise Co., LTD) were each diluted and applied at the recommended rate (Ministry of Agriculture, 2018) and at 100 times the recommended rate into each pot of 1 kg of bare soil. A ratio of 100 times the recommended dosage was chosen to represent the heavy and repetitive application scenario in the grape vineyard.

The water content of the bare soil was adjusted to 60% of the moisture content. The pots were pre-incubated for 2 weeks in darkness at 30 ± 2°C before pesticide applications to restore soil microcosms. The pesticide treatments included C (control soils), IM (0.0369 mg kg^−1^ and 3.69 mg kg^−1^), DT (0.0769 mg kg^−1^ and 7.69 mg kg^−1^), and ID (a combined mixture of IM 0.0369 mg kg^−1^ with DT 0.0769 mg kg^−1^ and IM 3.69 mg kg^−1^ with DT 7.69 mg kg^−1^). Each treatment had three replicates, giving a total of 24 containers. The control treatment was added with the same amount of deionized water to replace the pesticide application. Compensation for water loss during incubation for all treatments was done every 2 days with the addition of deionized water.

We conducted the experiments in two batches: high dosage (12 pots) and recommended dosage (12 pots). The pots were kept in a lab space with ambient room temperature and air moisture levels, so the variance between two batches of experiments and different sampling dates was expected.

### 2.3. Soil bacterial community-level physiological profiling analysis

Approximately 10 g of soil samples were taken from each pot at 0, 7, 14, 28, 56, and 63 days after pesticide treatments to determine the bacterial CLPP using the Biolog EcoPlate™ system (Biolog Inc., CA, USA). Before the analysis, 1 g of soil was serially diluted to 10^−3^ using a phosphate-buffered saline solution. Moreover, 130 μl of the above soil solution was transferred into each well of the Biolog EcoPlate to incubate it at 25°C for 72 h. Optical densities were observed at each 24-h interval at 590 nm and 750 nm, respectively (Classen et al., 2003). The average well-color development (AWCD), substrate richness (S), evenness (E), and Shannon diversity index (H') were calculated following the previous study (Zak et al., 1994).

### 2.4. Soil DNA extraction and next-generation sequencing

Soil microbial DNA extraction was performed using DNA Power Soil extraction kits (MO BIO Laboratories, Inc.) and stored at −20°C. DNA samples were sent to a company for NGS analysis (Genomics BioSci & Tech Ltd., Taiwan). The procedures were described in brief as follows: PCR amplifying the V3-V4 region of 16S rDNA using the primer set 341F-805R with the KAPA High-Fidelity PCR kit (KAPA BIOSYSTEMS) was performed. The products were purified using the QIAquick Gel Extraction Kit (QIAGEN). Afterward, the sequence libraries were generated using the Truseq nano DNA Library Prep Kit (Illumina, USA) and sequenced on an Illumina Miseq platform. Primer sequences were trimmed using the Cutadapt program and merged using FLASH software (v1.2.11). Mothur (v1.39.5) was used for picking operational taxonomic units (OTUs) with 97% identity. The OTU table was produced using UCHIME (v4.2) software.

### 2.5. Statistical analyses

The analysis of variance using a general linear model (multivariate) was performed at a significance level of *p*-value of < 0.05 by the least significant different *post*-*hoc* test (IBM SPSS Statistic version 25). Weighted variants of the UniFrac matrix were calculated and then visualized in the principal coordinates analysis (PCoA) using QIIME software (Bolyen et al., 2019).

## 3. Results and discussion

### 3.1. CLPP profiles and OTUs in the soils

**Figure 1:**
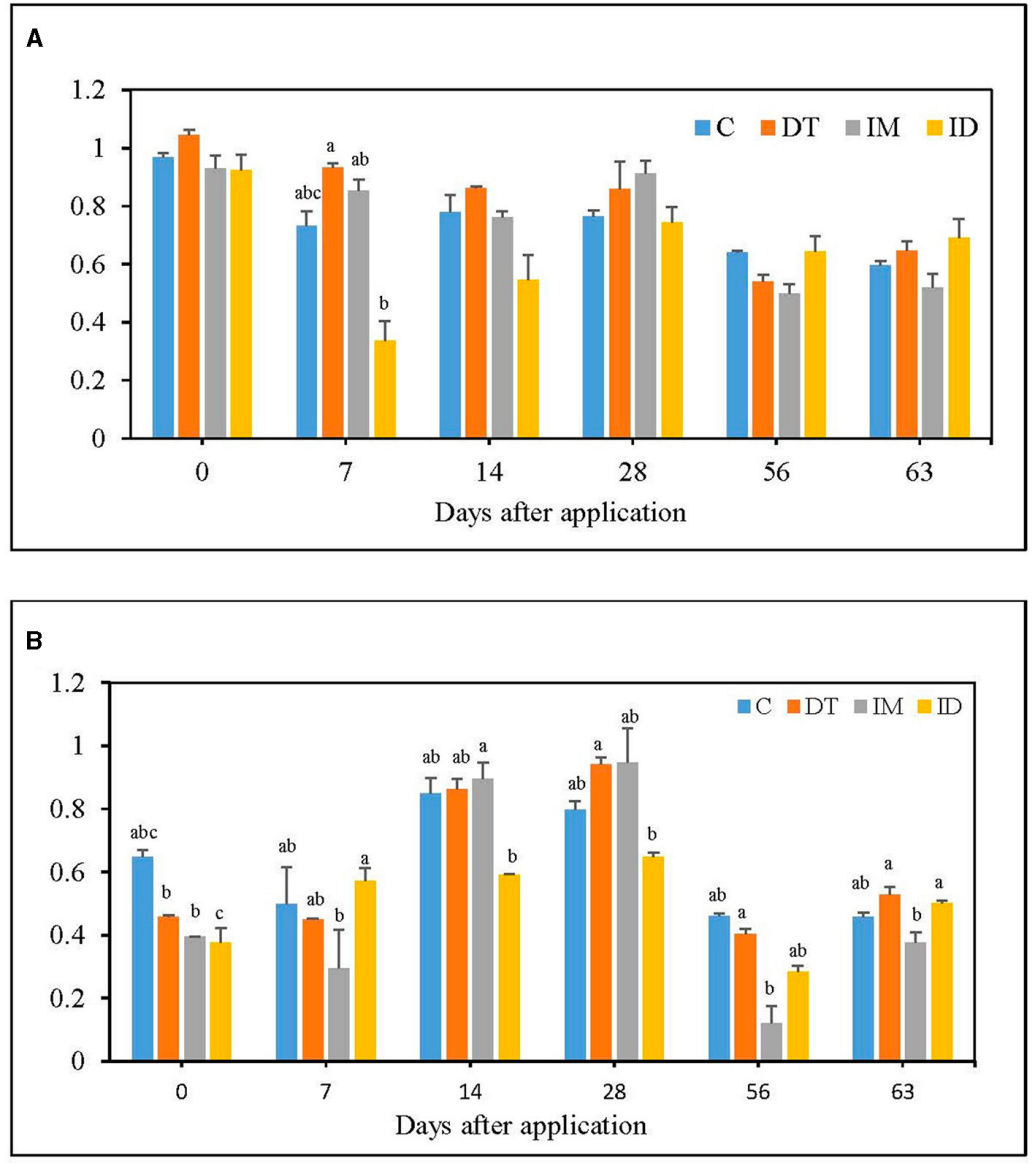
AWCD of EcoPlate **(A)** at the recommended dosage; **(B)** at 100 times the recommended dosage. The letters on the bars denote the difference in mean at a *p*-value of 0.05 in the LSD test.

**Figure 2:**
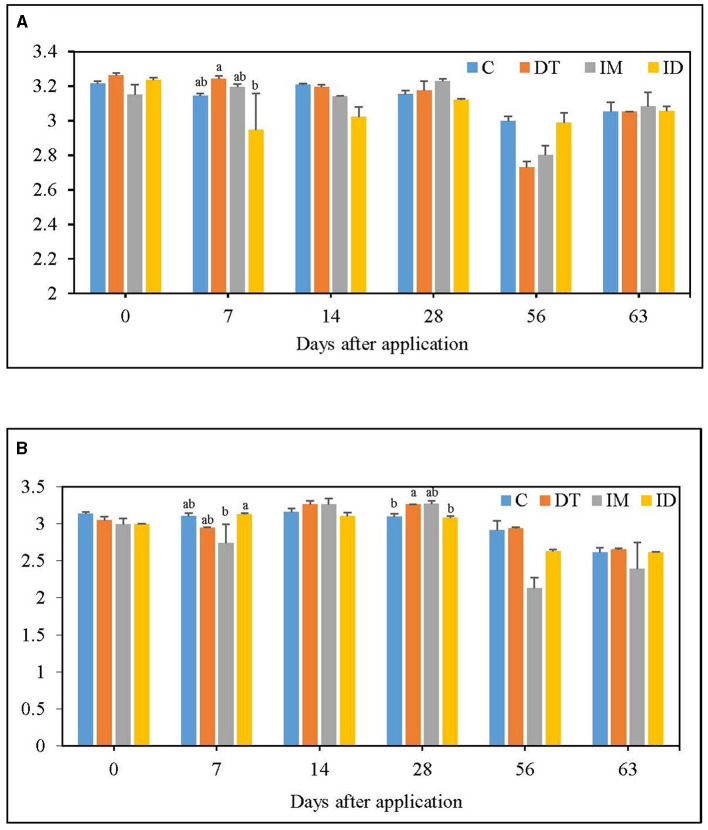
H index of EcoPlate **(A)** at the recommended dosage; **(B)** at 100 times the recommended dosage. The letters on the bars denote the difference in mean at a *p*-value of 0.05 in the LSD test.

The CLPP profiles were analyzed using the AWCD and Shannon diversity indices from EcoPlate™ results (Figures 1, 2). It is worth noting that this study's pot incubation was conducted in a lab space with ambient room temperature and air moisture level, so the variance was observed among different batches of experiments (recommended dosage group and high dosage group) and different sampling dates.

At the recommended dosage (Figure 1A), the AWCD level decreased upon co-exposure to dimethomorph and imidacloprid on day 7; however, on day 63, all treatments, including the combined treatment, were not significantly different from the control. A slight enhancement of soil metabolism was observed for dimethomorph and imidacloprid treatment on day 7; however, it was neither significant nor prolonged. This shows that, although the considerable effect on soil metabolism from co-exposure to pesticides may occur right after their application under the recommended dosage, soil metabolic activity could be restored within 2 months.

For the high dosage, imidacloprid slightly decreased average soil metabolism throughout the incubation period, showing its possibility of a prolonged effect (data not shown). However, the dimethomorph treatments did not alter soil metabolism during the 2 months of the incubation period (Figure 1B). As for the combined treatments of the two pesticides, inhibition of soil metabolism was observed on days 0, 14, and 28 but not on days 56 and 63. The results indicated that, under repetitive or high dosages, no significant or prolonged inhibition of soil metabolic activity was observed in the combined application either. We presumed that this could be due to the fact that dimethomorph can be more easily utilized by some soil microbes (Zhang et al., 2020) than imidacloprid or that the utilization of organic material released from dying fungal species was happening in the soil matrix (Katayama and Kuraishi, 1978) so that the effect of imidacloprid was concealed. A more pronounced decline of microbial metabolism by imidacloprid's separate applications under high dosage was recorded in our experiments rather than in the combined pesticides' applications.

A study reported that soil respiration was impeded by 100 mg kg^−1^ of dimethomorph but not by 1 or 10 mg kg^−1^ of that (Wang et al., 2017). In our study, 0.0769 mg kg^−1^ dimethomorph slightly increased AWCD in the short term, but within 2 months, it returned to the untreated state. Taking 100 times the recommended dose of dimethomorph could increase AWCD index on some sampling days, potentially obscuring the effects of other pesticides on soil.

Another study examined the soils treated with 50 mg kg^−1^ imidacloprid and found that the AWCD index was lower than the control (Garg et al., 2021). It was also reported that 1 mg kg^−1^ and 10 mg kg^−1^ of imidacloprid significantly decreased soil AWCD index (Cycoń et al., 2013). In our study, 0.0369 mg kg^−1^ of the imidacloprid did not significantly alter the soil metabolic activity, while 3.69 mg kg^−1^ of the imidacloprid could slightly decrease it. This shows that the effect of imidacloprid on soil metabolism could follow a dose-dependent pattern.

Fluctuations of bacterial diversity were observed at the recommended dosage for all treatments (Figure 2A); however, no significant difference was recorded from day 14 to day 63. A high dosage of dimethomorph increased bacterial diversity on day 28, while for imidacloprid, an inhibition was slightly shown on day 7 (Figure 2B). Nevertheless, regardless of whether the pesticide was administered at the recommended or high dosage, the influence of its individual and combined application was recovered within 2 months.

From NGS data (Table 1), an increase in OTU values can be observed from the combined treatments both at the recommended dosage and high dosage on day 7. However, the individual application of high-dose pesticides decreased the number of OTUs, but only in the short term. The application of dimethomorph and imidacloprid did not impose a significant difference after 2 months of examining the OTUs, which were in good accordance with the findings obtained from EcoPlates™.

**Table 1:** OTUs of treatments C, DT, IM, and ID on days 7 and 63.

	**Recommended dosage**				**High dosage**			
	**Day 7**		**Day 63**		**Day 7**		**Day 63**	
C	10,281.0 ± 1,466.9	b	21,140.3 ± 2,068.0	a	20,221.7 ± 1,714.6	ab	27,057.3 ± 723.4	a
DT	12,521.7 ± 814.1	b	17,570.3 ± 2,898.6	a	17,483.3 ± 940.3	b	23,930.3 ± 7,418.5	a
IM	15,308.3 ± 3,876.4	ab	16,240.3 ± 3,031.0	a	17,283.0 ± 2,764.1	b	30,994.7 ± 4,786.1	a
ID	18,529.0 ± 3,735.2	a	17,532.3 ± 1,803.8	a	23,681.3 ± 1,789.4	a	30,628.0 ± 1,212.5	a

Letters denote the difference in mean at a *p*-value of < 0.05 in the LSD test.

### 3.2. PCoA analysis of individual and co-exposure to pesticides on soil microbial taxa

**Figure 3:**
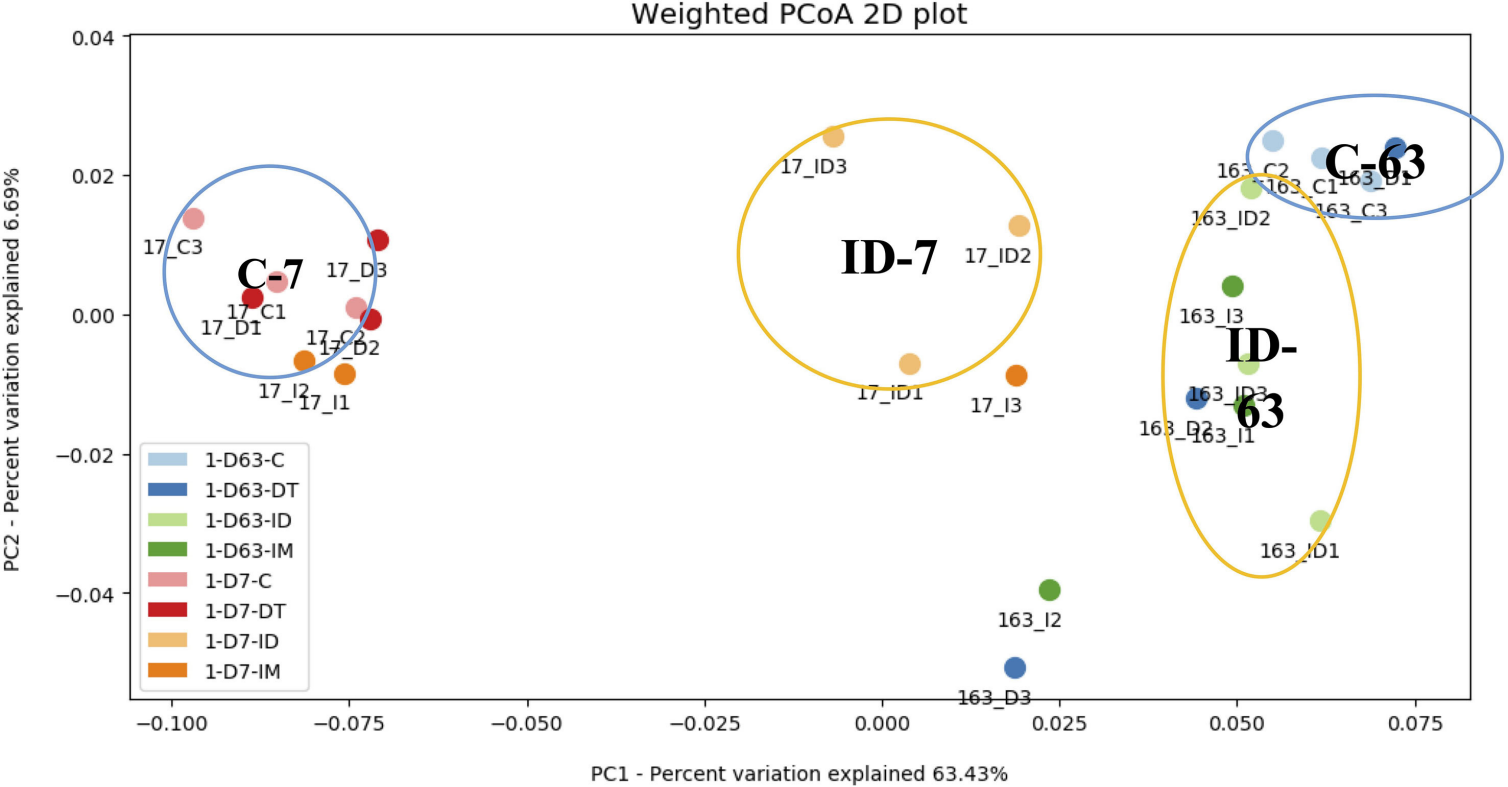
PCoA dendrogram of treatments C, DT, IM, and ID at the recommended dosage.

**Figure 4:**
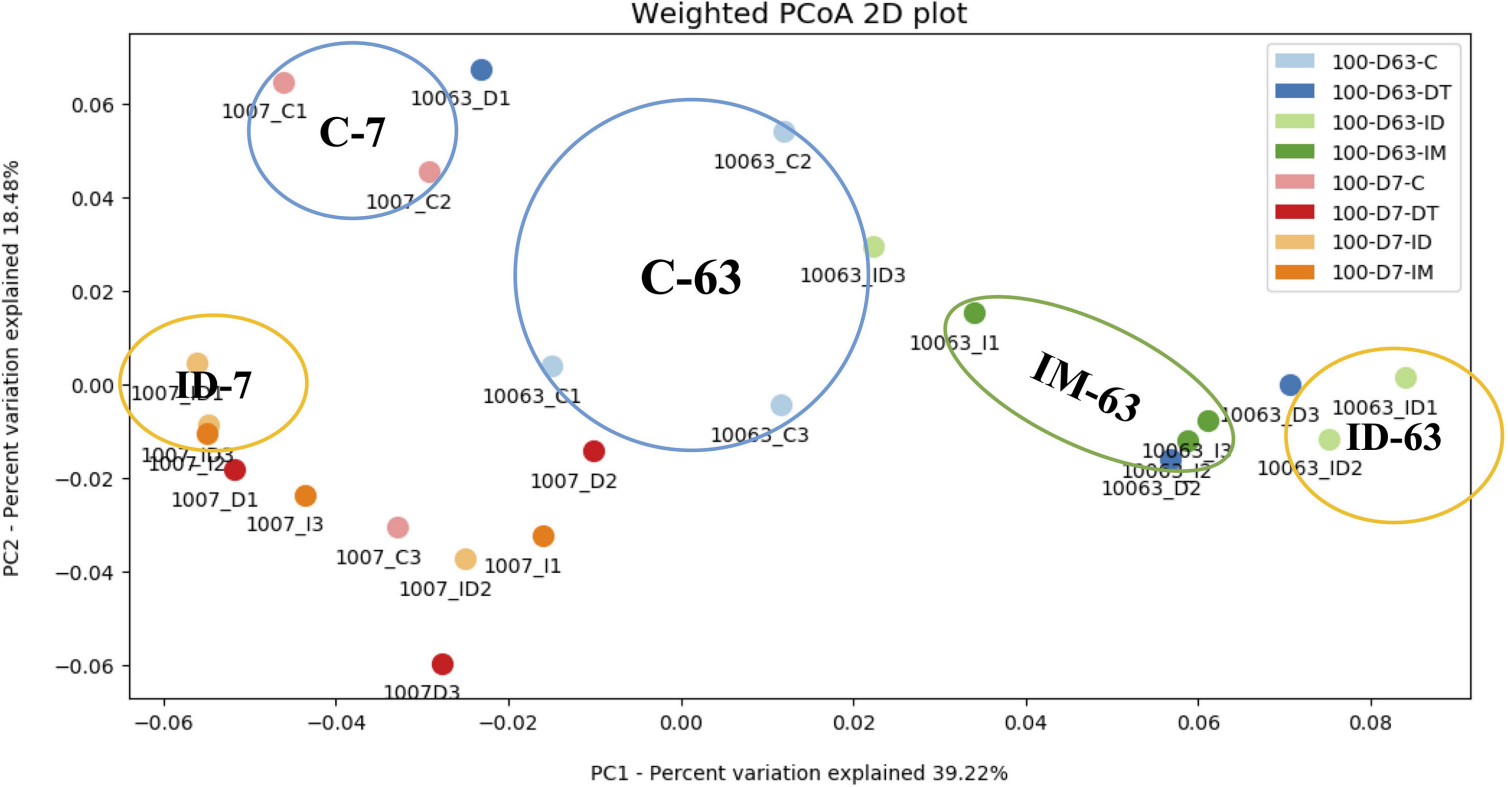
PCoA dendrogram of treatments C, DT, IM, and ID at high dosage.

To further evaluate the effect of individual and co-exposure to pesticides on soil bacterial communities, the similarity matrix of the bacterial species and abundance from different treatments were calculated and then visualized using PCoA dendrograms in Figures 3, 4.

At the recommended dosage (Figure 3), the control and individual treatments were both distant from the combined treatment ID on days 7 and 63. On day 7, the combined treatments were in a separate group from the individual treatments, while on day 63, the individual treatments of pesticides could be grouped with the combined treatments. The result indicates that, once treated with pesticides, regardless of whether it is a separate application or a combined application, the soil ecology would be divergent from the control after 2 months. However, under the recommended dosage, the more significant effect of combined treatments was only observed in the short term.

At high dosages (Figure 4), the control treatments on days 7 and 63 were distant from the pesticide treatments. It was observed that the combined treatments had exerted a more significant effect on the shifting of the bacterial ecology in soils throughout the incubation period since ID-7 and ID-63 were in unique groups distant from other treatments. In addition, the individual treatment of imidacloprid made a distinct cluster on day 63, showing a prolonged effect on the soil bacterial ecology.

Although studies have reported the shift in microbial metabolism and diversity under dimethomorph and imidacloprid separate applications (Cycoń et al., 2013; Wang et al., 2017), the effect of co-exposure to these two pesticides has not been reported. Our research found no evident long-term effect if examined only in soil metabolic activity, as elucidated in Section 2.1. If we consider both bacterial species and abundance data from NGS results, the possible long-term effect of pesticide application was revealed, whether in separate or combined application treatments.

In the following sections, we will examine the detailed change in the bacteria species, both at the phylum level and family level, to investigate the possible ecological effect of two pesticide applications.

### 3.3. Pesticide treatments on the bacteria phyla

Previous studies on the microbial biosphere have usually set 0.1 or 0.01% as the relative abundance threshold for rare taxa (Galand et al., 2009; Anderson et al., 2015) and 1% for abundant taxa within a sample (Galand et al., 2009; Liu et al., 2012). In this study, we focused on the examination of abundant taxa with a relative abundance >1%.

**Figure 5:**
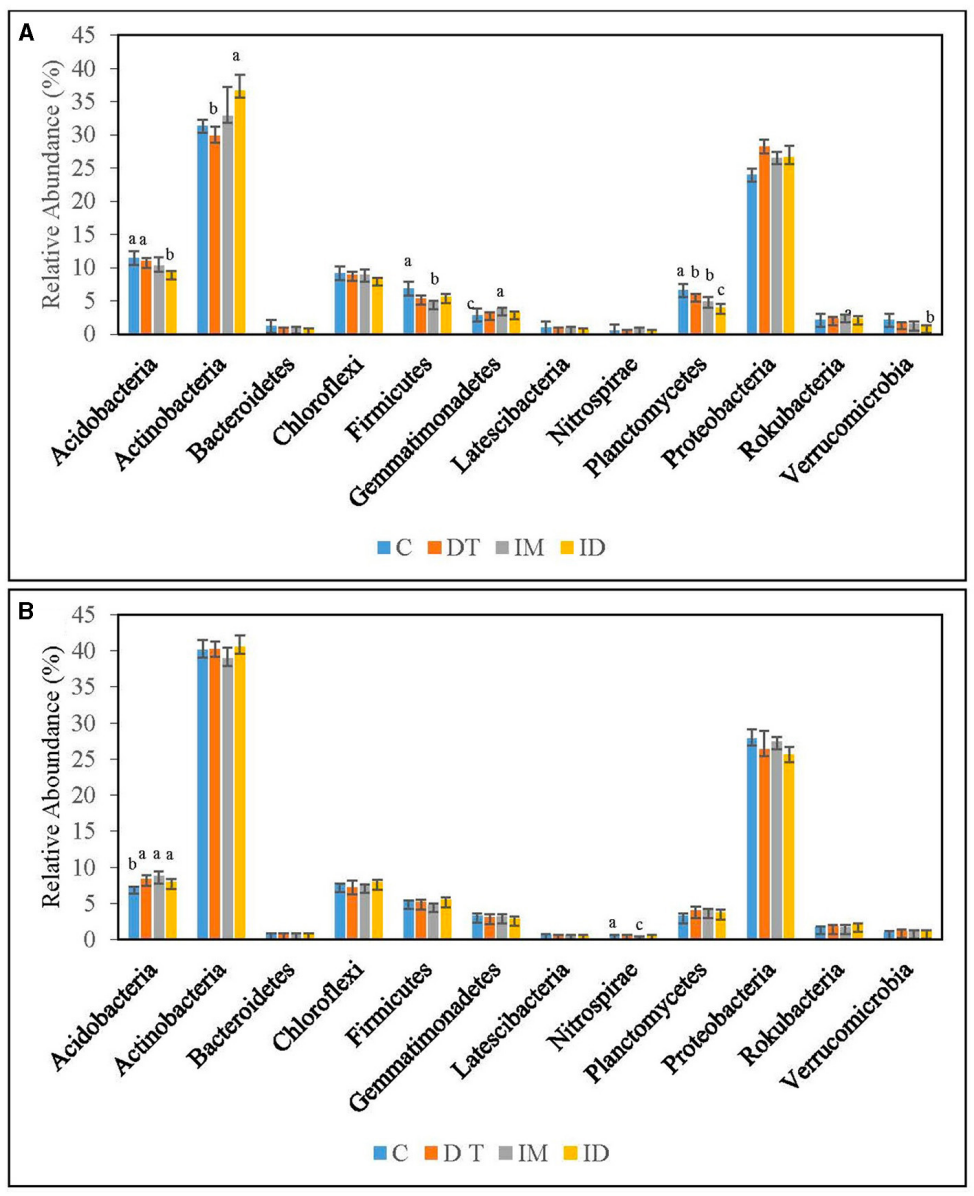
Bacterial phyla of treatments C, DT, IM, and ID at the recommended dosage on **(A)** days 7 and **(B)** 63. The letters on the bars denote the difference in mean at a *p*-value of 0.05 in the LSD test.

**Figure 6:**
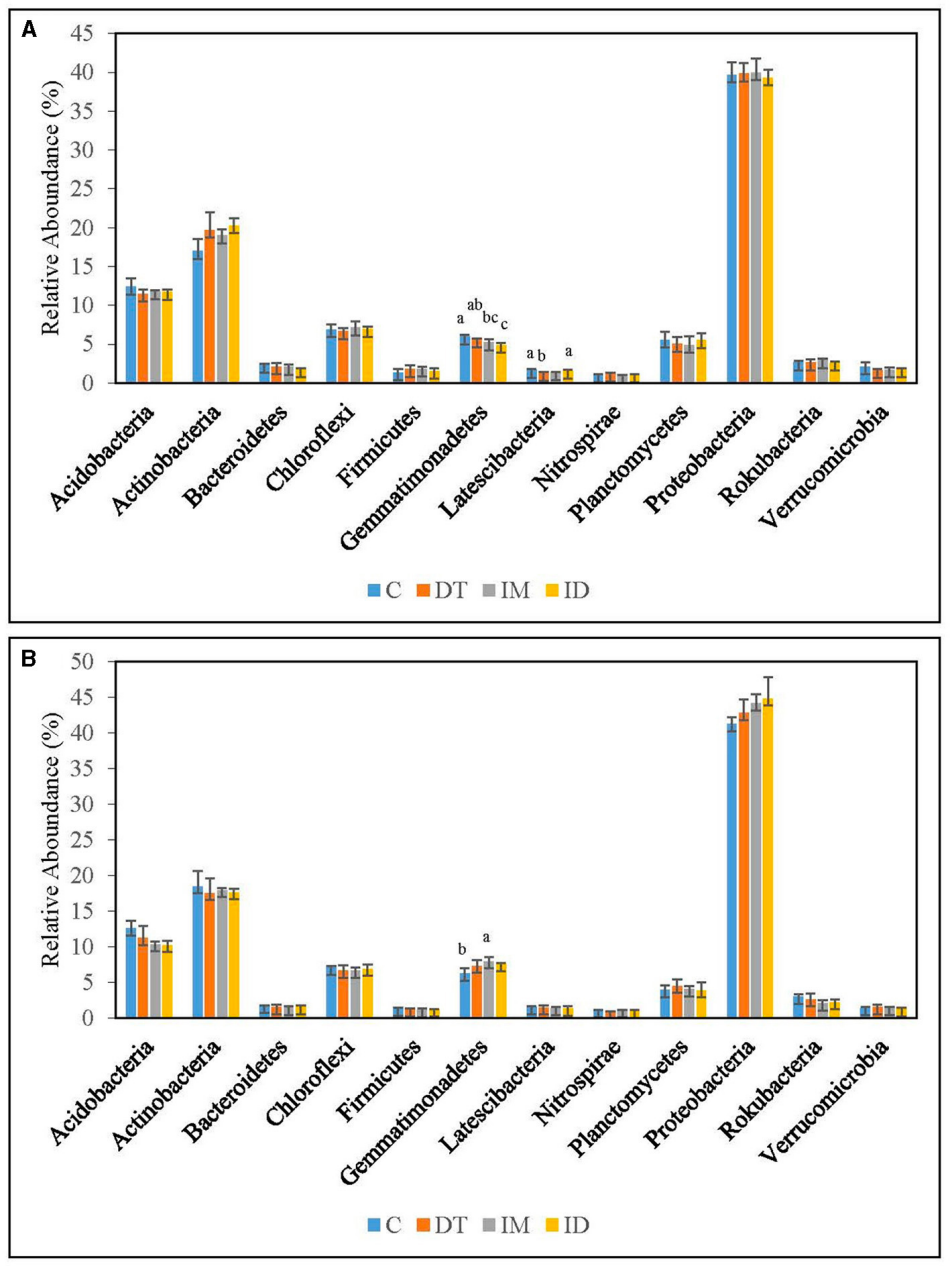
Bacterial phyla of treatments C, DT, IM, and ID at high dosage on **(A)** days 7 and **(B)** 63. The letters on the bars denote the difference in mean at a *p*-value of 0.05 in the LSD test.

The core phyla in the bare vineyard soil before and after imidacloprid and dimethomorph treatments were *Acidobacteria, Actinobacteria*, and *Proteobacteria* (Figures 5, 6). These three phyla accounted for 11.5, 31.3, and 24.0%, respectively, of the control treatments on day 7 (Figure 5A). It was previously reported that *Actinobacteria* and *Proteobacteria* phyla were the key taxa in two soil biomes in Brazil (Lupatini et al., 2014). *Acidobacteria* and *Proteobacteria* were also proposed as bacterial indicators for land-use change (Kim et al., 2021). Our findings are well-fitted with these previous studies.

Among the recommended dosage treatments (Figure 5), *Proteobacteria* were not altered significantly by the pesticide treatments. As for *Acidobacteria*, they were significantly decreased by the co-exposure of dimethomorph and imidacloprid on day 7 and was significantly increased by all pesticide treatments on day 63 (Figure 5). A previous study reported a shift in *Proteobacteria* and *Acidobacteria* by the change of soil pH and heavy metal pesticides (Kim et al., 2021). *Acidobacteria* were also reported to be augmented in fungicide-tebuconazole-contaminated soil (Baćmaga et al., 2022). In this study, we found that *Acidobacteria* were relatively sensitive to the effect of pesticides among these three abundant taxa when pesticide concentrations were around the recommended dosage. The phylum would be augmented once treated with dimethomorph and imidacloprid, either separately or combined.

Under high-dosage treatments (Figure 6), the minority soil species exhibited a more significant change than the core phyla, as no significant differences were recorded in the latter. *Gemmatimonadetes* was inhibited by ID on day 7; however, on day 63, it was significantly augmented by imidacloprid (Figure 6B). It was also found that the recommended dosage of imidacloprid had augmented it on day 7 (Figure 5A). We assumed that some members of *Gemmatimonadetes* might be able to utilize imidacloprid or its metabolites at a suitable concentration, although this has not been reported. *Gemmatimonadetes* comprise roughly 2% of soil bacterial communities, yet little is known of their ecology. The type strain of *Gemmatimonadetes* was *G. aurantiaca* strain T-27, a polyphosphate-accumulating isolate from wastewater (DeBruyn et al., 2011). Members of *Gemmatimonadetes* may probably serve as indicators upon imidacloprid application.

*Latescibacteria* can be inhibited by the high dosage of dimethomorph application but not by the co-exposure of pesticides on day 7 (Figure 6A). *Latescibacteria* was reported to be found in an aquifer contaminated with hydrocarbon and chlorinated solvents with protein-, lipid-, and polysaccharide-degradation abilities (Farag et al., 2017). The inhibition of *Latescibacteria* by the high dose of dimethomorph suggested a shift in soil functional genomics upon a high amount of dimethomorph application in the short term. Nevertheless, the co-exposure of pesticides also compensated for their ecological effect on soils.

The above results regarding the bacteria phylum indicated that the effect of co-exposure to pesticides would not be clearly observed compared with separate applications. For example, in Figure 5B, the boost of *Gemmatimonadetes* could only be observed in imidacloprid treatment, whereas in Figure 6A, the inhibition of *Latescibacteria* could only be observed in dimethomorph treatment. There were reports on the utilization of pesticides and their metabolites (Gangola et al., 2018a,b, 2022a,c, 2023; Bhatt et al., 2023) and on the fact that the microbes killed by biocides may become carbon and nitrogen sources of surviving microbes (Ullah and Dijkstra, 2019).

### 3.4. Pesticide treatments on the bacteria families at the recommended dosage

**Figure 7:**
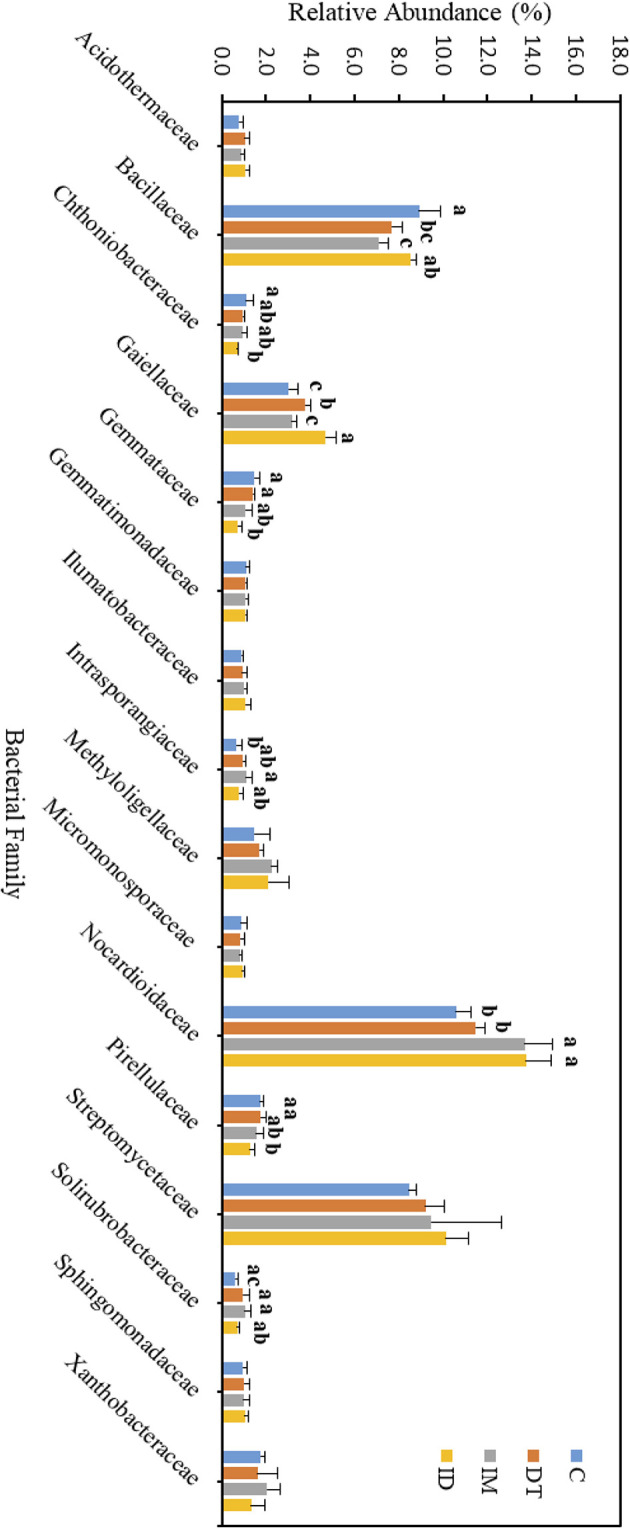
Bacterial family of treatments C, DT, IM, and ID at the recommended dosage on day 7. The letters on the bars denote the difference in mean at a *p*-value of 0.05 in the LSD test.

**Figure 8:**
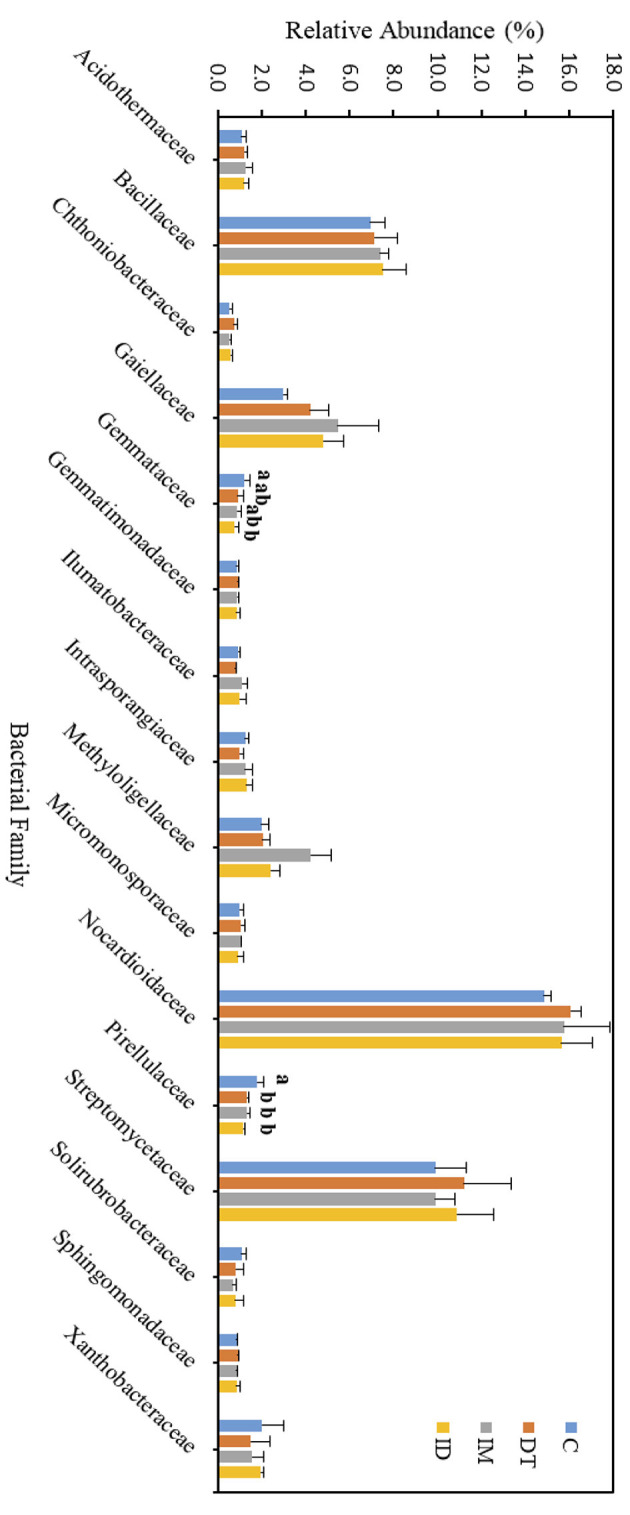
Bacterial family of treatments C, DT, IM, and ID at the recommended dosage on day 63. The letters on the bars denote the difference in mean at a *p*-value of 0.05 in the LSD test.

The core family members of the bare vineyard soil before and after the recommended dosage of pesticide applications were *Bacillaceae, Gaiellaceae, Nocardioidaceae*, and *Streptomycetaceae* (Figures 7, 8). Except for *Streptomycetaceae*, significant changes upon pesticide application were observed, but only on day 7.

On day 7, the relative abundance of *Bacillaceae* was significantly decreased by the individual application of dimethomorph and imidacloprid but not by the co-exposure of these two pesticides (Figure 7). This family comprises a large and diverse group of heterotrophic bacteria. It is known to participate in the carbon, nitrogen, sulfur, and phosphorous cycles in natural habitats (Mandic-Mulec et al., 2015). It was also reported that some bacteria belonging to this family had imidacloprid and other pesticide degradation abilities (Sabourmoghaddam et al., 2015; Gangola et al., 2021, 2023). Therefore, the degradation of pesticides in soils could also be impeded.

The bacterial family *Gaiellaceae* was significantly increased in DT and ID soil on day 7 (Figure 7). There is only one known strain, *Gaiella occulta*, in this family, which shows various abilities, including reducing soil nitrate (Albuquerque and da Costa, 2014). The augmentation in this family suggests that dimethomorph may enhance some ecological functions related to *Gaiellaceae*.

The relative abundance of the bacterial family *Nocardioidaceae* was significantly higher in the individual application of imidacloprid and in the co-exposure of dimethomorph and imidacloprid. Some members of *Nocardioidaceae* were active in the degradation of recalcitrant chemicals, such as phenols and nitrophenolic compounds, or toxic environmental pollutants and derivatives (Rosenberg et al., 2014; Tóth and Borsodi, 2014). The increase in the *Nocardioidaceae* abundance suggested the degradation of imidacloprid immediately after their application. A shift in pesticide degradation bacteria may also be observed from *Bacillaceae* to *Nocardioidaceae* at the recommended dosage.

Although the abundance change in the bacterial family can be observed among the core taxa on day 7, the above results show that if the recommended dosage was followed, the application of dimethomorph and imidacloprid, or the combined treatment, did not alter soil core bacterial families for more than 2 months or induce long-term reaction in the relevant carbon, nitrogen, sulfur, and phosphorous cycles, as well as the recalcitrant chemical degradative abilities related to those core families.

As for those bacterial families with lower abundances, *Chthoniobacteraceae, Gemmataceae*, and *Pirellulaceae* were significantly decreased by the co-exposure of pesticides but not the individual applications on day 7. After 2 months, we still observed a decline in the co-exposure treatment in *Gemmataceae*, while for *Pirellulaceae*, all the pesticide treatments would inhibit the abundance. These two families all belong to the *Planctomycetes* phylum. The *Planctomycetes* phylum is a unique heterotrophic free-living bacteria with a large genome and large genes, which might be responsive to the production of bioactive molecules (Wiegand et al., 2018).

*Gemmataceae* inhabit a wide variety of freshwater and terrestrial environments. Some of the members can utilize and degrade polysaccharides, chitin, and biopolymers, demonstrating their pronounced hydrolytic capabilities (Kulichevskaya et al., 2020). After 2 months, the decline in its abundance suggests that, even under the pesticide-recommended dosage treatments, the soil hydrolytic pathways toward those recalcitrant chemicals may still be impeded, and the co-exposure of pesticides may augment the effect.

As for the family *Pirellulaceae*, they can generally be found in fresh and marine water environments (Kulichevskaya et al., 2022). They were reported to be an ammonia-oxidizing bacteria, thus participating in the nitrogen cycle (de Celis et al., 2020). A previous study also showed that one species belonging to *Pirellulaceae* could be found in hexavalent chromium-contaminated garden soil, and after enrichment, the microbial consortia from the garden soil showed 99% of the chromium removal ability (Singh et al., 2022). After 2 months, the decline of its abundance suggests that pesticide application at the recommended dosage may also impede the nitrogen cycle and some contaminant removal abilities in soils.

### 3.5. Pesticide treatments on the bacteria families at high dosage

**Figure 9:**
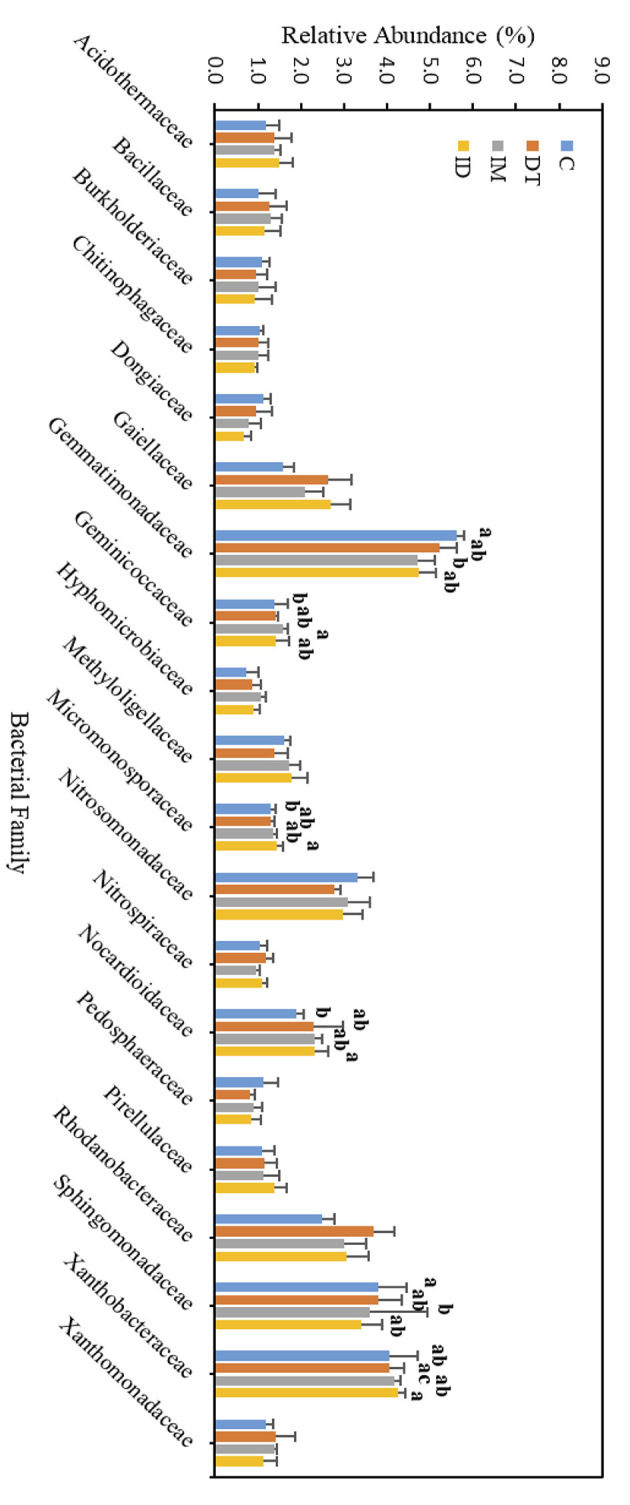
Bacterial family of treatments C, DT, IM, and ID at high dosages on day 7. The letters on the bars denote the difference in mean at a *p*-value of 0.05 in the LSD test.

**Figure 10:**
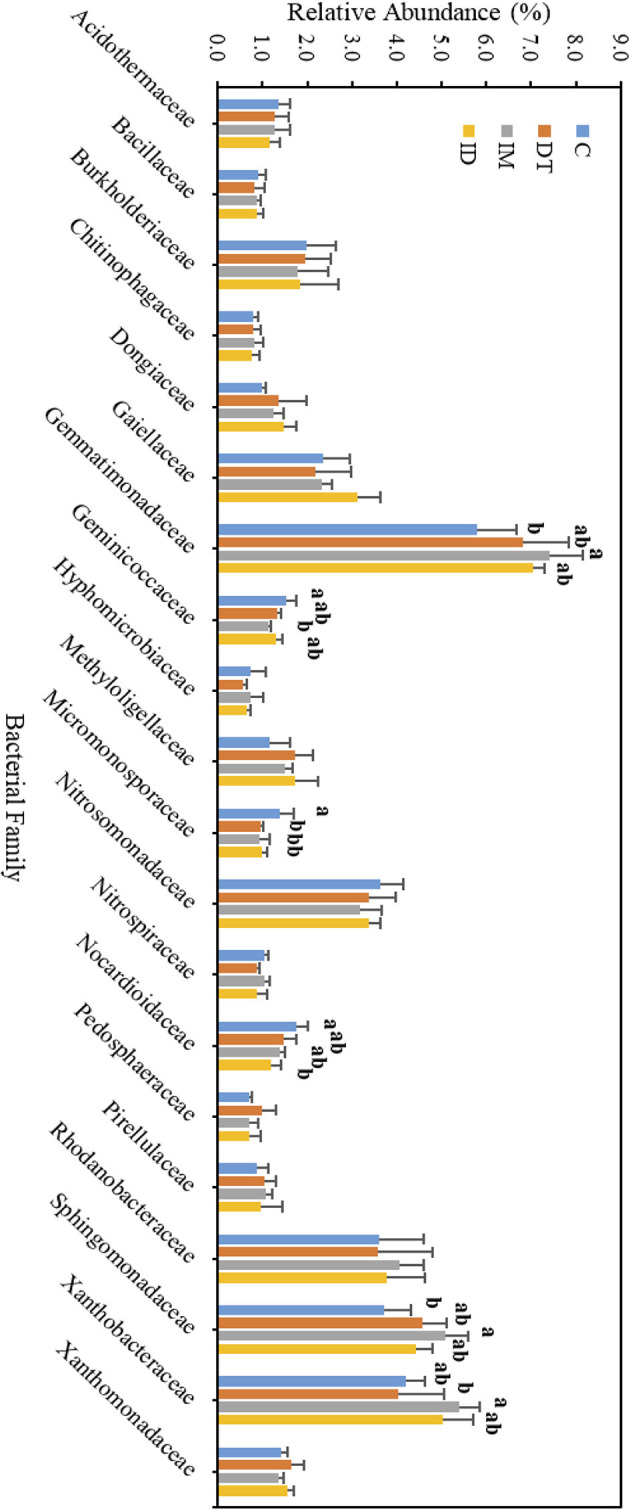
Bacterial family of treatments C, DT, IM, and ID at high dosages on day 63. The letters on the bars denote the difference in mean at a *p*-value of < 0.05 in the LSD test.

The core families of the bare vineyard soil before and after dimethomorph and imidacloprid treatments were *Gemmatimonadaceae, Nitrosommonadaceae, Nocardioidaceae, Phodanobacteraceae, Sphingomonadaceae*, and *Xanthobacteriaceae* throughout the incubation period (Figures 9, 10). Although the shift in the core families for the two batches of experiments (recommended dosage and high dosage) was observed, it should be noted that the room temperature and air moisture level also contributed to the difference, as described in Section 2.2.

Among the core families, the relative abundance of the bacterial family *Nocardioidaceae* was significantly augmented by the co-exposure of imidacloprid and dimethomorph treatment on day 7 (Figure 9) and was significantly inhibited by day 63 (Figure 10). This confirms that some members of *Nocardioidaceae* may utilize these pesticides, as we assumed in Section 3.4, but after 2 months, the lower substrate level may also limit their population.

Core family *Gemmatimonadaceae* was decreased by all pesticide treatments on day 7 (Figure 9); however, on day 63 (Figure 10), it was increased, especially by imidacloprid. *Gemmatimonadaceae* has only one type strain, *G. aurantiaca*, which grows slowly in activated sludge equipped with phosphate removal abilities (Hanada and Sekiguchi, 2014). It was also reported to have nitrate- and vanadium-reducing abilities (Jia et al., 2019; Fei et al., 2022). Other studies have reported that this family responded positively to nitrogen supply (Yuan et al., 2017). Relevant mechanisms related to *Gemmatimonadaceae* should be studied to reveal the effect of imidacloprid application.

Several species belonging to *Sphingomonadaceae* were reported to degrade xenobiotic and recalcitrant aromatic compounds of natural or anthropogenic origin (Glaeser and Kämpfer, 2014). This family was inhibited at the beginning of the imidacloprid application (Figure 9) but was augmented after 2 months (Figure 10).

Many species in *Xanthobacteraceae* were reported to be equipped with the nitrogen-fixing ability or the ability to grow on hydrocarbon substrates (Oren, 2014). The population was slightly augmented by imidacloprid and was slightly inhibited by dimethomorph after 2 months (Figure 10).

As for the lower abundance of bacterial families, the application of imidacloprid significantly increased *Geminicoccaceae*, while treatment ID significantly increased *Micromonosporaceae* on day 7 (Figure 9). However, on day 63, the two bacterial families were lower than the control (Figure 10). Some species belonging to the *Geminicoccaceae* family can have carbon dioxide fixation ability as well as starch and chitin degradation ability (Proença et al., 2018). These bacteria were also proposed to be sulfur-oxidizing bacteria (Vavourakis et al., 2019). The function of *Micromonosporaceae* in soil ecosystems is not well known. It was also reported that chitin had been used as a carbon source to isolate strains (Trujillo et al., 2014).

The above results showed that a high dosage or repeated application of imidacloprid can have a more significant effect on soil biological functions, including exotic aromatic carbon removal and the cycling of carbon, nitrogen, phosphate, and sulfur in the short term. However, the effect of pesticide application, regardless of whether it is a combined application or individual application, would be noticeable at the family level over a 2-month period.

## 4. Conclusion

In this present study, both EcoPlate™ and NGS data were used to analyze the effects of dimethomorph and imidacloprid on soil microbes and metabolic activity. Under the recommended dosage, the soil metabolic activity would have shown transient changes if we had examined soil metabolic activity only. Under repeated or high dosages, imidacloprid has a more significant inhibitory effect on soil metabolism, an impact that may be masked by the effects of dimethomorph. However, when bacterial species and abundance are considered jointly in the analysis, even at the recommended dosage, this leads to a divergence in overall soil ecology from the control group after 2 months, as shown in the PCA dendrogram—regardless of whether the pesticides are applied individually or in combination.

Even when considering species data, the effects of co-exposure to pesticides can still be hidden in some species. For example, the effect of imidacloprid on *Gemmatimonadetes* was immediately noticeable by the end of the incubation period. However, when dimethomorph was present, the adverse effects of imidacloprid were alleviated. We assume that the co-exposure of dimethomorph may provide the metabolites or the dead fungi as the carbon source in the soil; thus, the effect of individual applications of imidacloprid may be concealed. A similar compensation effect provided by imidacloprid to dimethomorph could also be observed. Nevertheless, further studies are urgently required for the combined application of pesticides with various metabolites and mechanisms to further reveal the possible mechanisms and ecological impact.

The results on bacterial families affected by pesticide applications in bare soil reflect changes in indigenous bacterial composition in the field. Changes in the composition of microbial communities would, in turn, affect the cycles of carbon, nitrogen, sulfur, and phosphorous, as well as contaminant removal ability within the vineyard. The pesticides used in the vineyard can threaten the natural environment by promoting the accumulation and migration of toxic substances in the ecosystem through weekly applications.

## Data availability statement

The original contributions presented in the study are included in the article/Supplementary material, further inquiries can be directed to the corresponding author.

## Author contributions

JC and W-CC conceived and designed the experiments. JC conducted the experiments, analyzed the data, and drafted the article. F-TS modified the experiments and analyzed the data. W-AL guided JC in some experiments. W-CC analyzed the data and wrote the article. C-SL helped to revise the article. All authors contributed to the article and approved the submitted version.
