# Supplementary material for: Co-exposure of dimethomorph and imidacloprid: effects on soil bacterial communities in vineyard soil
Source: Front Microbiol. 2023 Nov 2;14:1249167. doi: 10.3389/fmicb.2023.1249167 (PMC10653314; doi:10.3389/fmicb.2023.1249167)
Supplement: Supplementary file 1 [file Data_Sheet_1.PDF]

The co-exposure of dimethomorph and imidacloprid on soil bacterial communities in a vineyard soil

Jean Chang[a]†, Fo-Ting Shen[b]†, Wei-An Lai[b], Chien-Sen Lao[c], Wen-Ching Chen[d]*

[a] International Master Program in Agriculture, National Chung Hsing University, Taichung, Taiwan 40227

[b] Department of Soil and Environmental Science, National Chung Hsing University, Taichung, Taiwan 40227

[c] Department of Medical Science & Biotechnology, I-Shou University, Kaohsiung, Taiwan 82445

[d] International Bachelor Program in Agribusiness, National Chung Hsing University, Taichung, Taiwan 40227

Authors Information

† These authors share first authorship

*Corresponding author

Dr. Wen-Ching Chen (Associate Professor)

Address: International Bachelor Program in Agribusiness, College of Agriculture and Natural Resources, National Chung Hsing University

145 Xingda Rd., South Dist., Taichung City 402, Taiwan, R.O.C.

Tel.: +886-4-22840849 ext. 623

Fax: +886-4-22851922

E-mail: julychen@nchu.edu.tw

Table S1. Numbers of Genus, Species and the ecological roles of bacterial families in this study (part 1/3)

Family	Genus	Species	Ecological role	Citation
Acidothermaceae	1	1	Capable of carrying out rapid degradation of cellulose, at relatively high growth temperatures.	Berry, Barabote et al. 2014
Bacillaceae	86	>100	Saprophytes that participate in the carbon, nitrogen, sulfur, and phosphorous cycles in natural habitats.	Mandic-Mulec, Stefanic et al. 2015
Burkholderiaceae	23	>100	Capable of nitrogen fixation. Can degrade crude oils (including polycyclic aromatic compounds) and herbicides.	Coenye, 2014
Chthoniobacteraceae	not validly published		Precursors for the synthesis of vitamin K in decaying organic matter, important for plant growth.	Paterson, Adeleke et al. 2016
Chitinophagaceae	48	>100	Chitinophagaceae can degrade complex organic matters, such as chitin and cellulose (Rosenberg 2014), and show β-glucosidase activity (Bailey et al. 2013) an important enzyme associated with the breakdown of cellulose	Smith et.al. 2016
Gaiellaceae	1	1	Strictly aerobic and chemoorganotrophic. Assimilates a few sugars, as well as myo-inositol, organic acids, and amino acids.	Albuquerque and Da Costa 2014
Gemmataceae	5	5	Relevant to phosphorous removal from wastewater.	Mahajan, 2019
Geminicoccaceae	4	5	Sulfur-oxiding bacteria	Vavourakis, Mehrshad et al. 2019
Gemmatimonadaceae	2	3	Hyphomicrobiaceae and Gemmatimonadaceae were also closely related to nitrogen removal.	Jia et.al. 2019
Hyphomicrobiaceae	12	40	Removal of odorous volatile sulfur compounds such as H_2S, methane thiol and dimethylsulfide from air and gasses.	Oren and Xu, 2014
Intrasporangiaceae	24	77	Biological phosphorus removal from wastewater.	Rosenberg, DeLong et al. 2014
Ilumatobacteraceae	2	4	Aerobic chemoorganotrophic bacteria	Coll, Bier et al. 2020

Table S1. Numbers of Genus, Species and the ecological roles of bacterial families in this study (part 2/3)

Family	Genus	Species	Ecological role	Citation
Methyloligellaceae	not validly published		MPs enriched this bacteria family, possible role in carbon (C) cycling within the paddy field.	Ghashghavi, Hester et al. 2019 Feng, He et al. 2020
Micromonosporaceae	37	>100	Degrade chitin, cellulose, lignin, and pectin, and these microorganisms play an important role in the turnover of organic plant material. Treating diabetes and made for antibiotics.	Trujillo, Hong et al. 2014
Nitrosomonadaceae	3	9	Play major roles in control of the nitrogen cycle in terrestrial, freshwater, and marine environments and in wastewater treatment processes.	Prosser, Head et al. 2014
Nocardioidaceae	5	>100	Ability to metabolize recalcitrant and complex compounds, including toxic environmental pollutants, alkanes of various lengths, crude oils, and derivatives. There are reports on the ability of members of this genus to degrade phenols and nitrophenolic compounds.	Rosenberg, DeLong et al. 2014
Nitrospiraceae	3	9	The family is physiologically highly diverse and contains chemolithoautotrophic aerobic nitrite-oxidizing bacteria (Nitrospira), chemolithoautotrophic aerobic and acidophilic ferrous iron oxidizers (Leptospirillum), and anaerobic, thermophilic, chemoorganoheterotrophic or hydrogenotrophic sulfate reducers (Thermodesulfovibrio).	Holger, 2014
Pedosphaeraceae	1	1	The enriched families of Pedosphaeraceae, were positively correlated with metal concentration in plants.	Cao, Luo et al. 2020
Phycisphaeraceae	2	2	All known species are Gram-stain negative and show binary fission. Endospores are not observed. Motile. Aerobic and acultatively anaerobic. Oxidase-negative, catalase-positive.	Fukunaga, Kurahashi et al. 2009

Table S1. Numbers of Genus, Species and the ecological roles of bacterial families in this study (part 3/3)

Family	Genus	Species	Ecological role	Citation
Pirellulaceae	7	15	Ammonia-oxidizing bacteria, plays a role in nitrogen cycle.	Miao, Wang et al. 2019
Rhodanobacteraceae	15	71	Members of the Rhodanobacteraceae family (close to clone 4) have also been described as able to mineralize BaP (Kanaly et al., 2002), as shown by the production of 14CO2 from 14C labelled BaP on a high-boiling-point diesel fuel distillate (HBD). BaP solubilisation into the culture suspension was however an essential step for rapid mineralization. In the case of our consortium, the solubilisation could occur thanks to biosurfactant producing bacteria, making the degradation of BaP possible. One of PAHs degradation bacterias.	Cazals et al. 2020
Streptomycetaceae	14	> 100	Able to degrade complex and fractious animal and plant materials.	Lindsay and Dyson, 2014
Steroidobacteraceae	2	4	May exhibit reduction of nitrate to dinitrogen monoxide and further to dinitrogen or ammonia (assimilation).	Liu, Liu et al. 2019
Sphingomonadaceae	24	> 100	Candidates for bioremediation of wastewater or contaminated dumping sites. Sphingomonas strain sp. FG03 isolated from phenolcontaminated soils was positively tested for phenol degradation.	Glaeser and Kämpfer, 2014
Xanthobacteraceae	9	35	May play an important role in the degradation of toxic organic compounds in polluted environments.	Oren, 2014
Xanthomonadaceae	not validly published		Xanthomonadaceae are capable of utilizing cellulose during the wood decomposition process and play a role in carbon turnover (Herve et al., 2014).	Saddler and Bradbury, 2015<br>Qiu et. al. 2019
